# Traction-Free Arthroscopy Using an Inside-Out Technique for Treating Femoroacetabular Impingement Syndrome

**DOI:** 10.1016/j.eats.2025.103589

**Published:** 2025-05-22

**Authors:** Lingchao Ye, Xiangang Jin, Huixia Fan, Qingguo Zhang, Pangtao Chen, Yongzhi Ye, Dawei Han, Li Ying, Junbo Liang, Xiaobo Zhou

**Affiliations:** Department of Orthopedics, Taizhou Hospital of Zhejiang Province affiliated to Wenzhou Medical University, Linhai City, Zhejiang, China

## Abstract

Hip arthroscopy is widely used for treating conditions such as femoroacetabular impingement (FAI) and labral tears. Traditionally, traction has been considered essential during hip arthroscopy to provide adequate working space for procedures in the central and peripheral compartments. However, perineal posts, commonly used with traction boots, have been associated with iatrogenic injuries, including perineal nerve damage. Although modified traction-free techniques have been introduced, they still carry risks of traction-related complications. To address these limitations, we developed a traction-free hip arthroscopy technique designed to minimize the risk of traction-related injuries while maintaining procedural efficacy. This approach reduces exposure to fluoroscopic radiation for both patients and surgeons, enhancing safety and efficiency during hip arthroscopy.

With the growing awareness of hip pathologies and advancements in surgical techniques, the number of hip arthroscopy procedures has significantly increased in recent years. During these surgeries, most surgeons rely on a perineal post to counteract the sustained traction applied to the lower limb in the supine position.^1^^,^^2^ However, prolonged perineal compression from the post can lead to complications such as soft-tissue damage (e.g., scrotal necrosis and labial injury) or neurologic injuries, including perineal pain and erectile dysfunction.^3^^,^^4^

Hip arthroscopy technology is relatively complex and involves a long learning curve. Regardless of the operator's proficiency, traction may cause surgical complications. A retrospective study involving 218 adolescent hip arthroscopies reported a complication rate of 1.8%, with nerve injuries accounting for 50% of cases. Similarly, a systematic review indicated that the rate of complications from traction-assisted hip arthroscopy using a perineal post can reach 7.1%.^5^^,^^6^ To mitigate these risks, researchers have developed traction-free techniques such as the "Tutankhamun technique" proposed by Salas et al.^7^ Although this technique eliminates the perineal post, its complexity and the excessive restriction of the upper extremities may interfere with anesthesia and intravenous fluid administration. Currently, the simplification of the surgical process and avoidance of surgical complications caused by traction remain important clinical issues. To address these challenges, we propose a traction-free hip arthroscopy method that can be performed using conventional surgical beds.

## Surgical Technique

### Preoperative Planning

A comprehensive preoperative assessment should be performed, including a detailed history, physical examination, and imaging studies. Physical examination should evaluate range of motion, hip strength, and specific tests such as flexion, adduction, and internal rotation, as well as flexion, abduction, and external rotation. The flexion, adduction, and internal rotation test demonstrates a sensitivity of 99% in diagnosing femoroacetabular impingement. Imaging studies, particularly unilateral hip magnetic resonance imaging, are essential for assessing labral, cartilaginous, and other soft-tissue injuries.

### Positioning and Anesthesia

**Fig 1:**
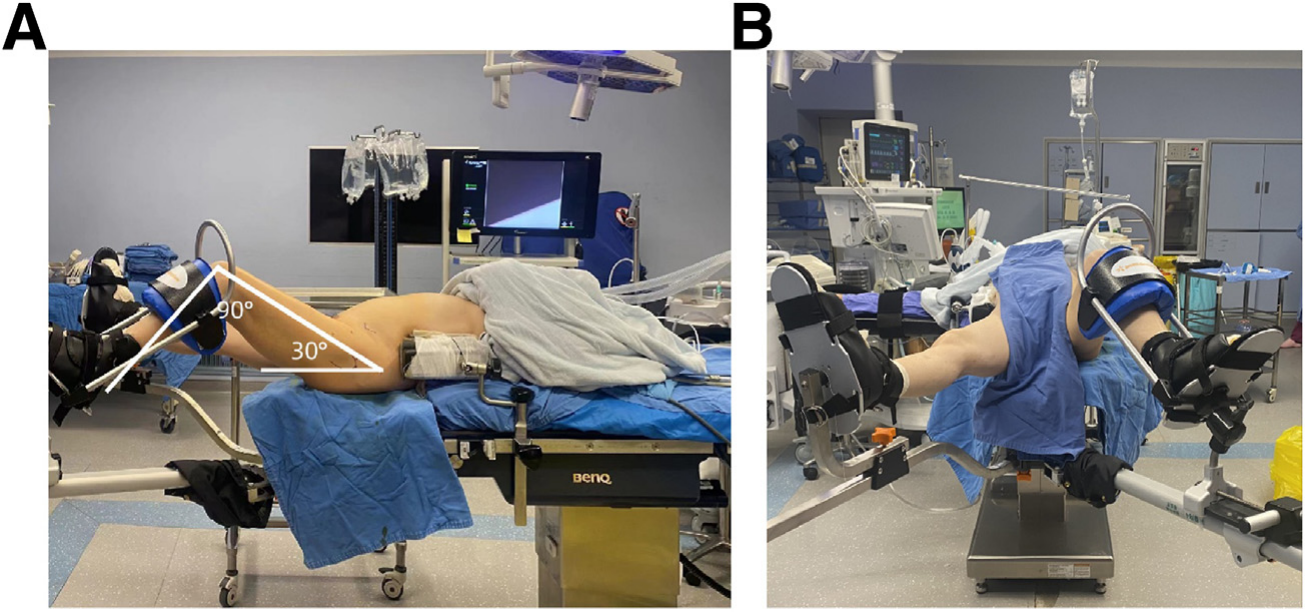
Patient positioned supine on a traction-free surgical table, exposing the left hip joint. (A) The patient’s left hip is positioned in 30° flexion and 90° knee flexion. (B) The patient’s left hip is positioned in 45° abduction and 15° internal rotation.

**Fig 2:**
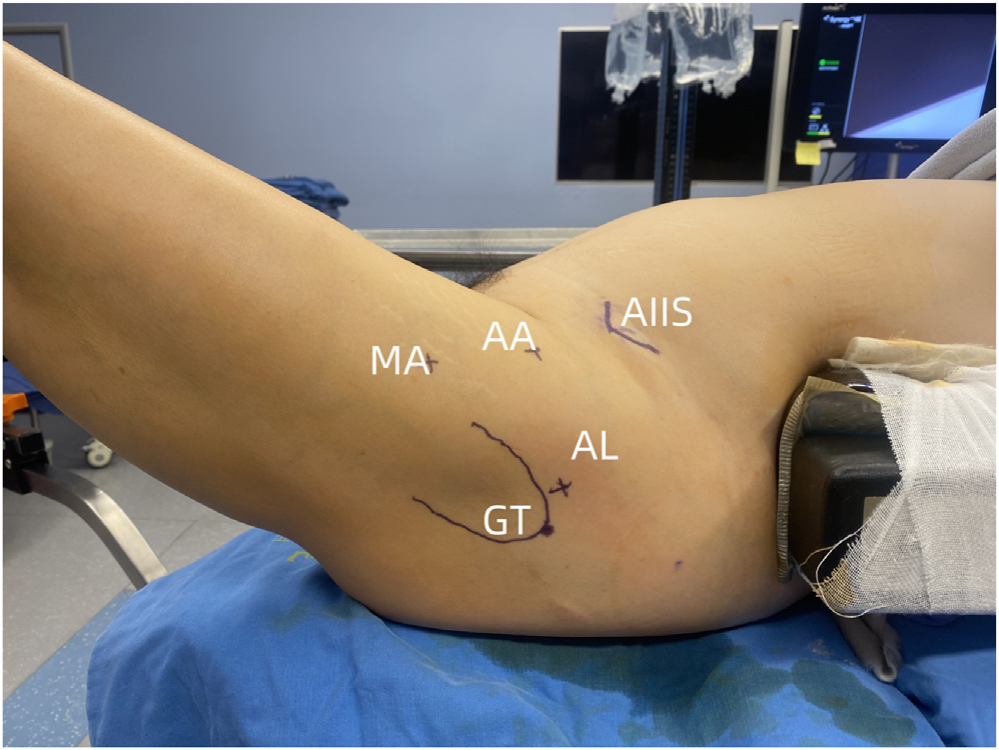
Arthroscopic portals for the left hip: anterolateral (AL) portal, midanterior (MA) portal, and anterior (AA) portal. (AIIS, anterior inferior iliac spine; GT, greater trochanter.)

The procedure is performed with the patient in a supine position using a traction-free surgical table (Smith & Nephew). After the induction of general anesthesia, soft jelly pads are placed bilaterally on the iliac crests to stabilize the patient's trunk (Fig 1). The surgical limb is positioned at 90° knee flexion, 30° hip flexion, and 15° internal rotation, and then secured using a padded boot (Fig 1). The surgical site should be prepared and draped in a standard sterile fashion. Bony landmarks and planned arthroscopic portals should be marked on the skin (Fig 2).

### Portal Establishment and Capsulotomy

**Fig 3:**
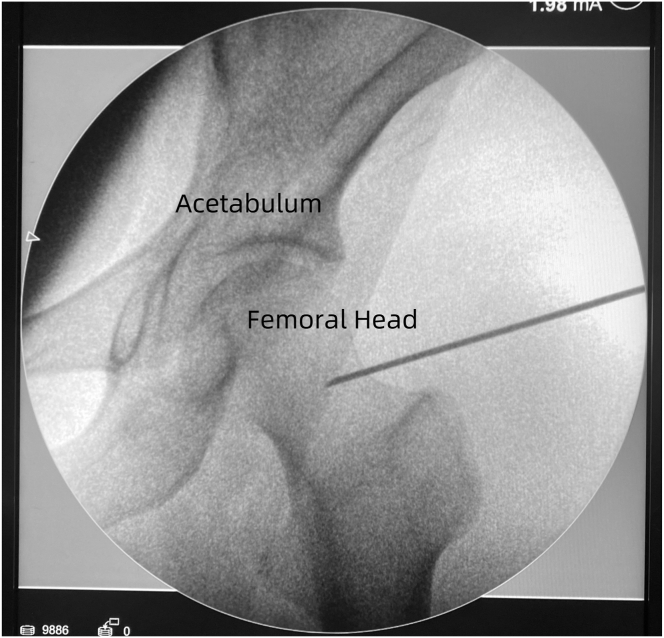
Fluoroscopic confirmation of the spinal needle position. The optimal entry point is at the femoral head-neck junction, just below the ligamentum teres, perpendicular to the femoral neck, with the needle tip sliding past the anterior edge of the femoral neck.

The anterolateral portal (AL) is created 2 cm anterior to the tip of the greater trochanter, directed distally at 30° inclination and 15° anteversion. A spinal needle is used to pierce the joint capsule at the anterolateral corner of the femoral neck, confirmed fluoroscopically (Siemens Healthineers) (optimal entry: just below the ligamentum teres attachment at the femoral head–neck junction) (Fig 3). A cannula is inserted to establish a viewing portal, and a 30° arthroscope is introduced with saline infusion to create an operative space (Smith & Nephew Endoscopy).

The midanterior portal is established 5 cm distal and 2 cm anterior to the AL portal under direct visualization. The spinal needle is introduced into the capsular space parallel to the viewing portal and positioned 0.5 to 1.0 cm distal to the AL portal, guided fluoroscopically if necessary, for novice surgeons. A cannula is inserted to create the working portal (Video 1).

**Fig 4:**
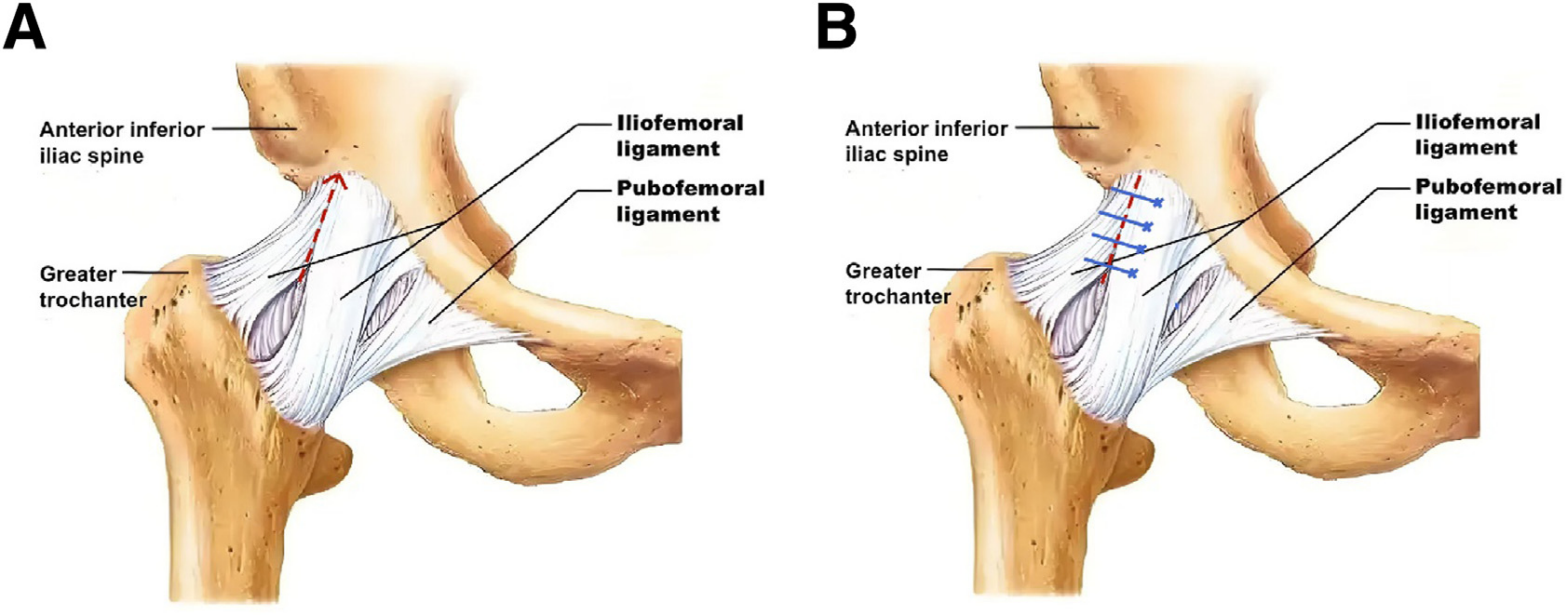
Schematic diagram of joint capsule incision. (A) Cut the joint capsule along the posterior bundle of the iliofemoral ligament. (B) Standard joint capsule closure is performed using edge to edge suturing method. Red dashed line, cutting direction; Blue solid line, stitching direction.

**Fig 5:**
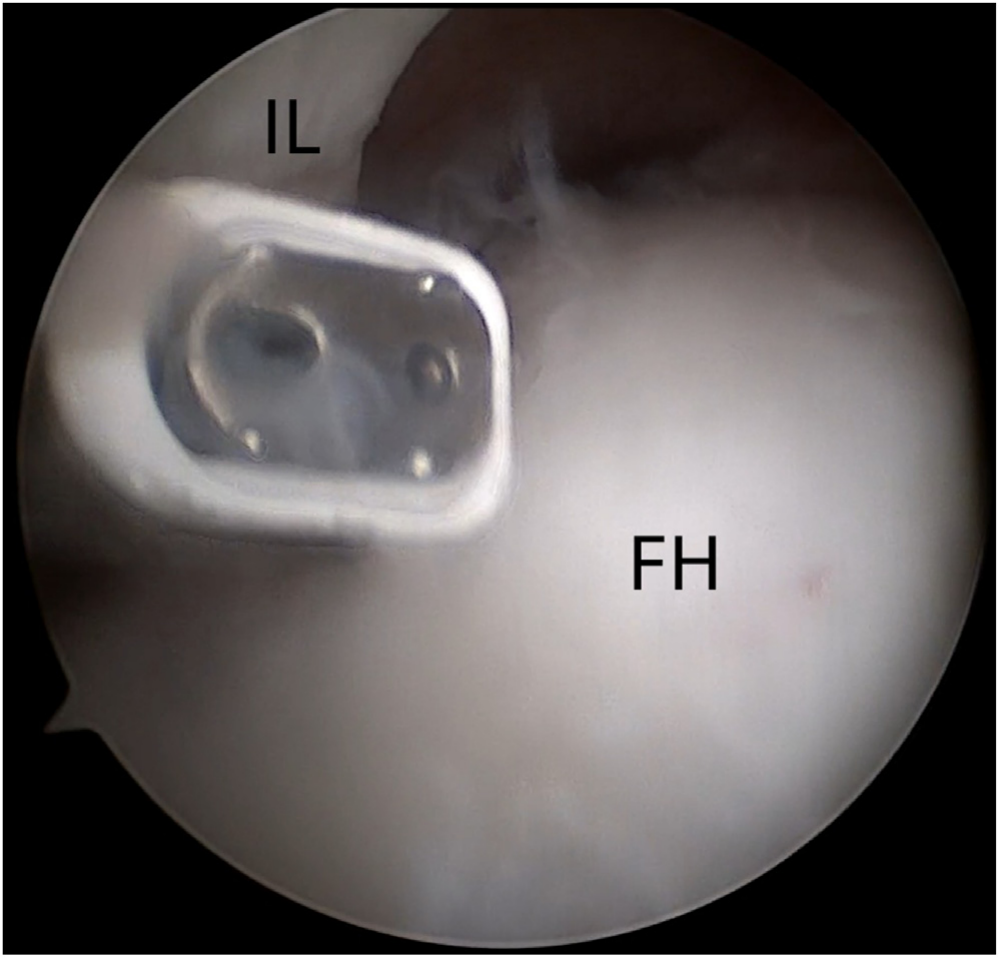
Arthroscopic view of the intra-articular space of the left hip joint using a 30° arthroscope via the anterolateral portal. Capsulotomy is performed from inside out. (FH, femoral head; IL, iliofemoral ligament.)

A 90° radiofrequency probe (DePuy Synthes, Johnson & Johnson) is used to perform a longitudinal capsulotomy along the posterior bundle of the iliofemoral ligament, extending distally from the joint interior to the exterior. The observation portal is then switched to the mid-anterior portal, and the anterolateral portal is repurposed as the working portal. Capsulotomy is extended proximally toward the inferior border of the anterior inferior iliac spine (Figs 4 and 5).

**Fig 6:**
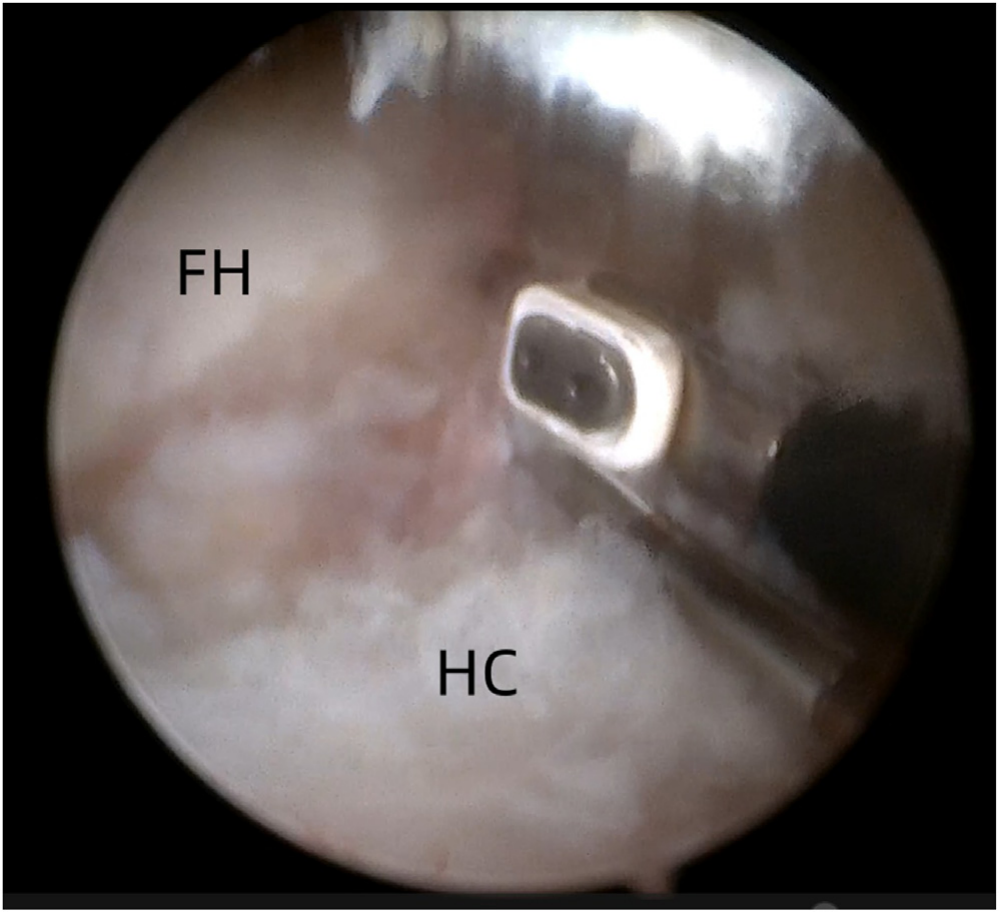
Arthroscopic view of the intra-articular space of the left hip joint using a 30° arthroscope via the anterolateral portal. The anterior portal is established. (FH, femoral head; HC, hip capsule.)

An additional anterior portal (A) is created at an equilateral triangular point from the AL and midanterior portals, 4 cm inferior and 3 cm lateral to the anterior inferior iliac spine. Under direct visualization, the spinal needle is advanced into the joint capsule anterior to the femoral head (Fig 6).

### Labral Repair and Femoral Head Osteoplasty

**Fig 7:**
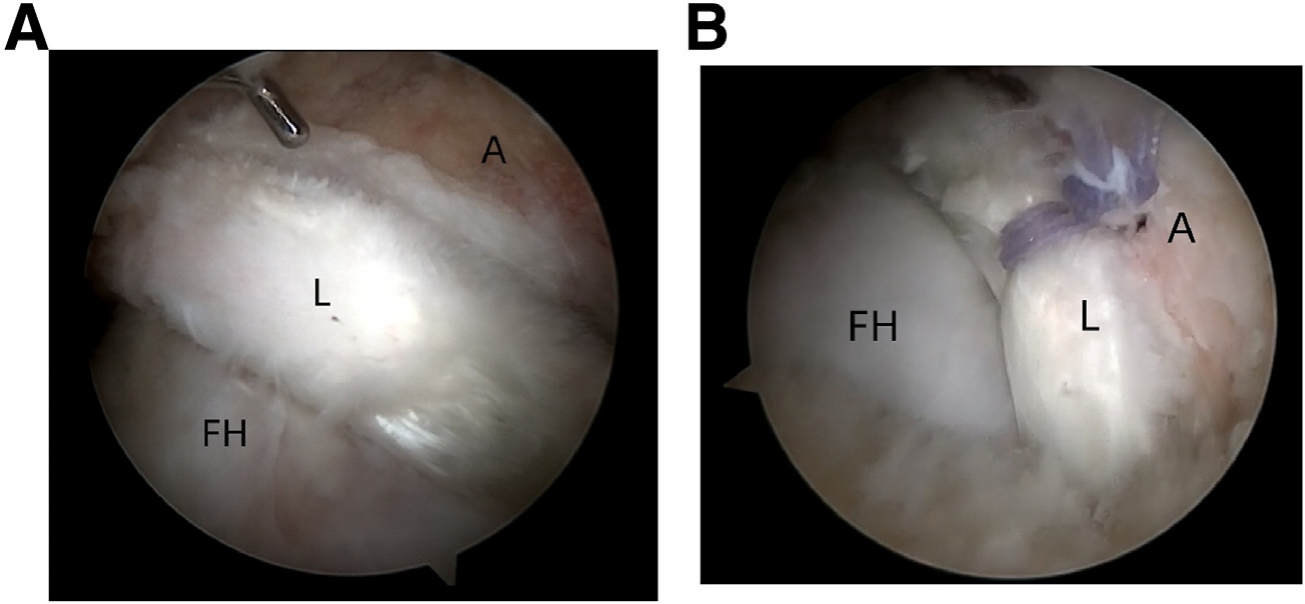
Arthroscopic view of the intra-articular space of the left hip joint using a 30° arthroscope via the anterolateral portal (A). (A) Acetabuloplasty performed with a motorized burr. (B) Circumferential labral repair. (FH, femoral head; L, labrum.)

The femoral head cartilage and acetabular labrum also should be assessed, and labral tears should be repaired whenever feasible. Damaged labral tissues are identified and prepared on the basis of preoperative imaging and intraoperative findings. Acetabular rim trimming is performed using a motorized burr (Smith & Nephew), and 2 to 4 suture anchors (DePuy Synthes, Johnson & Johnson) are placed for labral refixation. Labral repair is completed using knot-tying sutures and the loop-stitch technique (Fig 7, Video 1).

**Fig 8:**
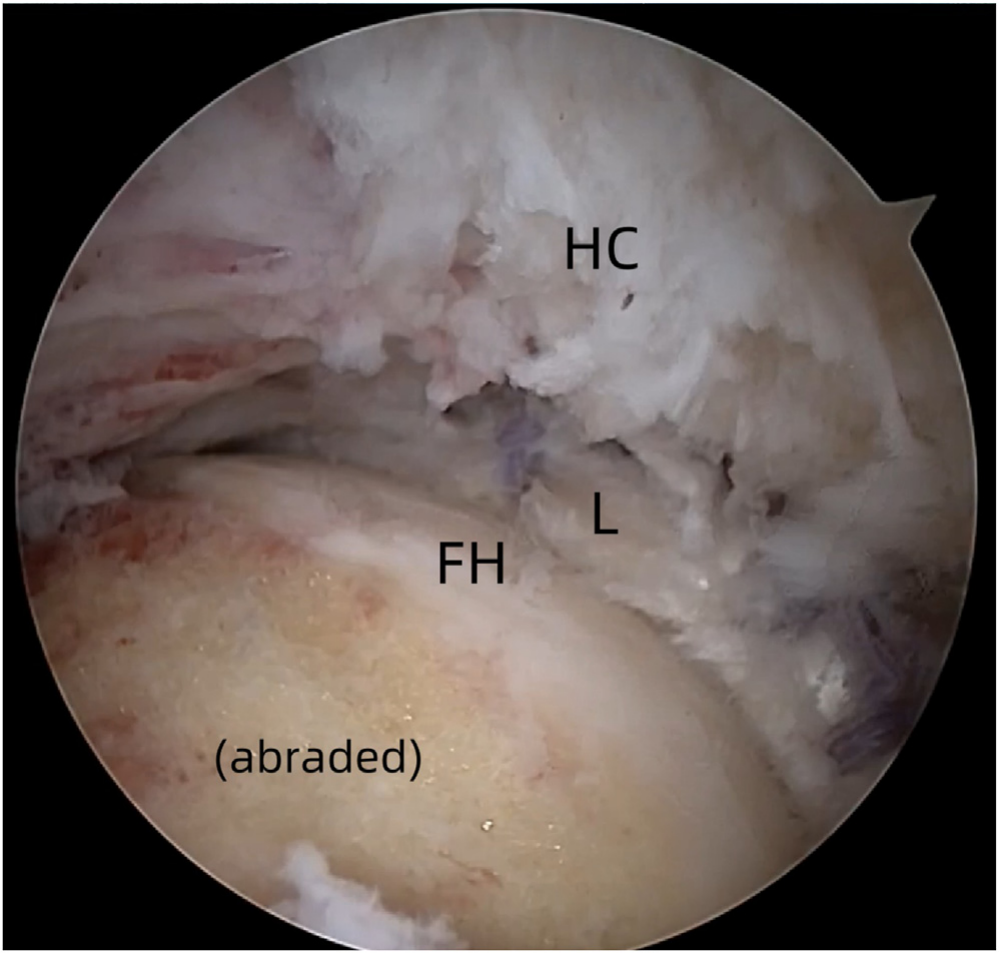
Arthroscopic view of the intra-articular space of the left hip joint using a 30° arthroscope via the anterolateral portal. (FH, femoral head, L, labrum, HC, hip capsule.)

**Fig 9:**
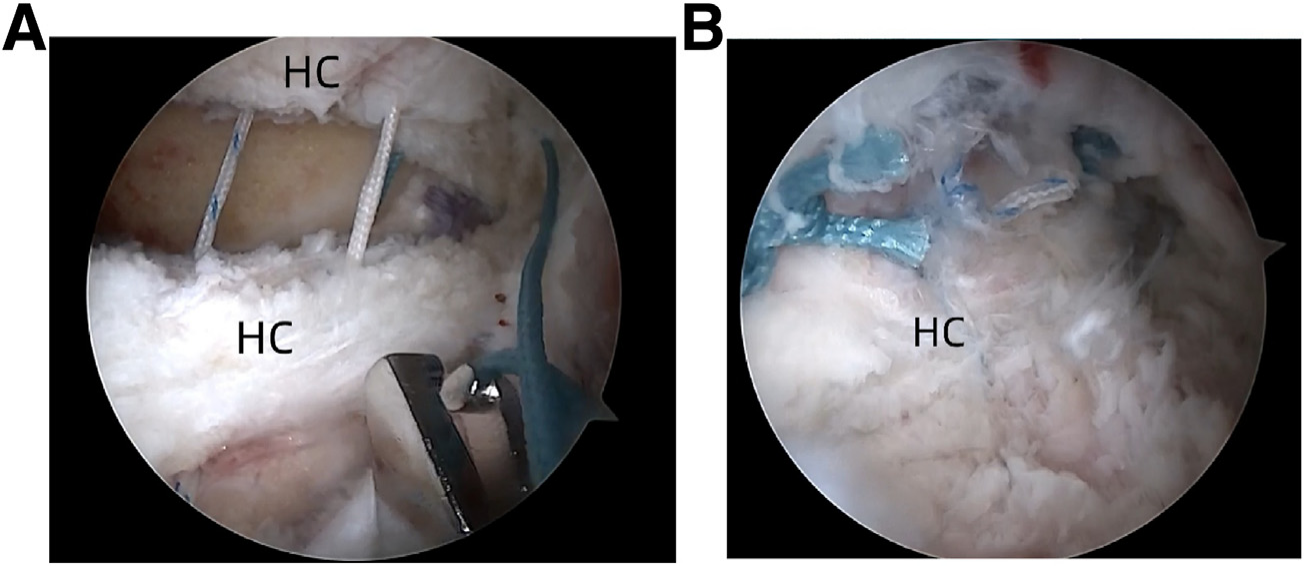
Arthroscopic view of the extra-articular space of the left hip joint using a 30° arthroscope via the anterolateral portal. Standard capsular closure performed using a side-to-side suture technique. (A) Before suture. (B) After suture. (HC, hip capsule.)

Femoral head osteoplasty is performed with a motorized burr to address cam deformities (Smith & Nephew). Bone resection continues until the transition zone between the sclerotic and cancellous bones is achieved, ensuring a uniform surface with mild bleeding. Range-of-motion testing can be used to confirm the adequacy of the osteoplasty (Fig 8). Postoperative capsular closure is performed using high-tensile, nonabsorbable sutures (e.g., Smith & Nephew or ETHIBOND sutures) with a side-to-side technique (Fig 9).

### Postoperative Rehabilitation

Patients should be encouraged to bear weight as tolerated within the first 2 weeks postoperatively. Full hip mobility is typically regained by 6 weeks, and light sports activities can be resumed by 12 weeks postsurgery.

## Discussion

Hip arthroscopy technique has been continuously innovated in recent years^8^^,^^9^ and is widely used in the treatment of hip joint conditions, including acetabular impingement and labral tears.^10^ Conventional hip arthroscopy often employs perineal post-assisted traction in the supine position. However, prolonged perineal compression associated with the use of perineal stents can lead to complications such as circulatory damage and persistent tissue ischemia.^11^ To address these issues, researchers have proposed postless distraction techniques that avoid the risks of nerve damage associated with perineal post traction. For example, Wang et al.^12^ proposed seat belt and abdominal belt techniques, while Salas et al.^13^ proposed yoga mat techniques. Studies have shown that traction-free hip arthroscopy could result in shorter hospital stays for patients.^14^ However, these techniques are still associated with challenges such as insufficient counterforces leading to intraoperative instability and residual risks of traction-related neurovascular damage.

To address these limitations, we propose the "traction-free hip arthroscopy" technique, which enhances the surgical field of view by flexing the hip and knees. This technique eliminates the need for hip joint traction, thereby completely avoiding traction-associated risks. Furthermore, it ensures patient safety by preventing slipping off the operating table without relying on perineal post traction. The horizontal supine positioning reduces the cardiovascular burden and minimizes the risk of gastroesophageal reflux associated with tilting the surgical table.^15^

This technique also minimizes radiation exposure, as intraoperative fluoroscopy is limited to the initial portal establishment, with subsequent steps performed under direct visualization. The flexed hip and knee positioning improve anterior joint space visualization and facilitate femoral head osteoplasty. Key procedures such as chondroplasty, labral repair, and acetabular rim trimming can be effectively performed using this approach. For labral injuries located deep within the joint cavity, auxiliary traction can be used if necessary, although this is rarely required.

This technique exhibits risks and limitations that warrant consideration. First, variability in surgeons' technical proficiency may lead to unintended tissue damage during channel establishment. Second, the absence of hip joint traction results in a narrower surgical field of view compared to traditional arthroscopy, potentially complicating the detection of lesions such as posterior labral tears. Third, the technique may be less effective for complex pathologies like diffuse synovial chondromatosis, where comprehensive visualization and access are critical. Lastly, the requirement for extended joint capsule dissection to compensate for lack of traction necessitates meticulous suturing to maintain structural integrity (Table 1). However, our experience suggests that traction-free hip arthroscopy is effective for managing a vast majority of hip conditions. This approach avoids traction-related complications, preserves the joint capsule, requires no additional equipment, and is straightforward to learn, making it a valuable addition to hip arthroscopy.

**Table 1:** Advantages and Limitations of the Traction-Free Hip Arthroscopy Technique

Advantages	Limitations
Eliminates the risk of traction-related surgical complications	Establishing the initial surgical portals can be challenging
Reduces intraoperative fluoroscopy usage, minimizing radiation exposure for patients and medical staff	May limit surgical procedures for certain specific conditions
Flexed hip and knee positioning increases anterior visualization space, facilitating femoral head osteoplasty	Narrower surgical field of view may result in missing damaged areas
	A meticulous focus on capsular suture integrity is imperative at the conclusion of the procedure to ensure long-term structural stability.

## Disclosures

All authors (L.Y., X.J., H.F., Q.Z., P.C., Y.Y., D.H., L.Y., J.L., X.Z.) declare that they have no known competing financial interests or personal relationships that could have appeared to influence the work reported in this paper.

## Supplementary Data

Video 1 In this video, we demonstrate a traction-free hip arthroscopy technique. The patient lies supine on a standard column free hip joint traction table, placing their feet in cushioned boots. Then, the patient's lower limb on the surgical side is bent 90°, the hip is bent 30°, and the leg is fixed in an internal rotation position of 15°. Mark the bone markers and plan the entrance under arthroscopy. The anterior lateral approach (AL) is located at the anterior inferior iliac spine, 2 cm in front of the apex of the greater trochanter of the femur. The middle anterior approach (MA) is located 5 cm distal to the AL approach, and about 2 cm forward. The anterior approach (A) is located about 4 cm downward and 3 cm outward from the anterior inferior iliac spine. At this point, the 3 surgical approaches are connected to form a pattern similar to an equilateral triangle. After establishing the operating space, operations such as acetabular shaping, labrum repair, and femoral head shaping can be completed under the microscope. After the surgery is completed, standard joint capsule closure can be performed using edge to edge suturing.
